# Clinical Outcomes of Fixed Dental Prostheses Supported by Teeth or Implants in the Posterior Region—A Long‐Term Retrospective Multicenter Study

**DOI:** 10.1111/clr.70169

**Published:** 2026-08-22

**Authors:** Zahra Athab Abduljabbar, Lars Hjalmarsson, Victoria Franke Stenport, Jir Barzangi, Anders Magnuson, Alf Eliasson

**Affiliations:** ^1^ Department of Prosthodontics/Dental Materials Science, Institute of Odontology, Sahlgrenska Academy Gothenburg University Gothenburg Sweden; ^2^ Center for Clinical Research Sörmland Uppsala University Eskilstuna Sweden; ^3^ Specialist Dental Clinic, Folktandvården Sörmland AB Mälar Hospital Eskilstuna Sweden; ^4^ Brånemark clinic, University Dental Care, Public dental service Region of Västra Götaland Sweden; ^5^ Center for Clinical Research, Västmanland County Hospital Uppsala University Västerås Sweden; ^6^ Clinical Epidemiology and Biostatistics, Faculty of Medicine and Health, School of Medical Sciences Örebro University Örebro Sweden; ^7^ Dental Research Department, Faculty of Medicine and Health Örebro University Örebro Sweden

**Keywords:** biological complications, dental implant, fixed dental prostheses, survival, technical complications

## Abstract

**Background:**

Few long‐term studies compare tooth‐ and implant‐supported fixed dental prostheses (FDPs) in the posterior region.

**Objectives:**

To compare the 5‐ and 10‐year survival and complication outcomes of tooth‐ and implant‐supported FDPs and to assess patient‐reported effects.

**Material and Methods:**

This retrospective multicenter clinical study investigates patients treated with 3–5 units tooth‐supported fixed dental prostheses (TSFDPs) and 1–4 units implant‐supported FDPs in the posterior region at two specialist dental clinics (2009–2019) using clinical and radiographical examinations and Oral Health Impact Profile‐14 (OHIP‐14). Survival, biological, and technical complications as well as patient‐reported effects were recorded.

**Results:**

A total of 180 patients with 238 FDPs: 113 TSFDPs, 71 implant‐supported crowns (ISCs), and 54 implant‐supported fixed partial prostheses (ISFPPs) were examined after a mean follow‐up of 10 years. The 5‐year cumulative survival rate for TSFDPs and implant‐supported FDPs was 98% (*p* = 0.71). The 10‐year cumulative survival rates were 94% for TSFDPs and 93% for implant‐supported FDPs (*p* = 0.87). Eight TSFDPs, three ISCs, and five ISFPPs failed. Biological complications were recorded in 24.8% of TSFDPs and in 9.6% of implant‐supported FDPs (*p* = 0.012). Technical complications were recorded in 29.9% of TSFDPs and in 27% of implant‐supported FDPs (*p* = 0.62). Low OHIP‐14 scores were reported in patients with tooth‐supported (4.8 ± 6.7), implant‐supported (3 ± 4.3), and with both types of FDPs (3.5 ± 3.6). Scores that indicate a good OHRQoL.

**Conclusion:**

Tooth‐ and implant‐supported FDPs had high survival rates after 5 and 10 years. Biological complications had the highest impact on prostheses failure.

## 1 Introduction

For many years, single or multiple missing teeth in the posterior region of the dental arch were replaced with tooth‐supported fixed dental prostheses (TSFDPs) (Creugers et al. 1994; Rabel et al. 2024). However, TSFDP increases risk for endodontic complications as it requires reduction of the abutment teeth (Hebel et al. 2000; Pjetursson et al. 2007). A review study reported that complications in TSFDPs were observed in 15.7% of patients (Pjetursson et al. 2007). The most frequently reported technical complications were loss of retention and fracture of the veneering material (chipping) (Alsterstal‐Englund et al. 2021; Hawthan et al. 2023; Pjetursson et al. 2015). The main causes for the loss of or modification of TSFDPs were caries, endodontic complications, loss of retention, root fracture, and framework fracture (Alsterstal‐Englund et al. 2021; Glantz et al. 1993; Hawthan et al. 2023; Palmqvist and Swartz 1993; Randow et al. 1986). However, implant‐supported fixed dental prostheses to replace single or multiple missing teeth in the posterior dental arch could solve these problems without reduction of tooth substance (Hebel et al. 2000; Pjetursson et al. 2018; Rabel et al. 2024; Sailer et al. 2018). Several studies of implant‐supported crowns (ISCs) and implant‐supported fixed partial protheses (ISFPPs) reported an implant survival rate > 95% after 5–10 years (Hjalmarsson et al. 2016; Jung et al. 2012; Pjetursson et al. 2007, 2012). Despite the high implant survival rate, complications were reported in 33.6%–38.7% of patients with ISFPPs after 5 years (Pjetursson et al. 2007, 2012). The most frequent complications with ISCs and ISFPPs were abutment screw loosening, veneer fracture, mucositis, peri‐implantitis, loss of screw‐access hole restoration, and loss of retention of cemented prostheses (Jung et al. 2012; Pjetursson et al. 2012). In addition, abutment fracture, screw fracture, and implant fracture were also reported (Eliasson et al. 2006; Kim et al. 2010; Rabel et al. 2018; Sailer et al. 2022). When making treatment decisions regarding tooth‐ or implant‐supported fixed dental prostheses, the key question is which prosthetic rehabilitation that has the best prognosis and least risk for complications in the specific situation. Some studies have compared survival and complication rates between tooth‐ and implant‐supported fixed dental prostheses in the posterior region (Bischof et al. 2024; Pjetursson et al. 2007; Walton 2015; Wolleb et al. 2012). However, few long‐term outcome studies compared the two prosthetic treatment alternatives (Bischof et al. 2024; Ermatinger et al. 2024; Pol et al. 2022; Salinas and Eckert 2007; Walton 2015). Furthermore, not enough evidence supports the idea that implant‐supported fixed dental prostheses are superior to TSFDPs regarding patient‐reported effects (Pol et al. 2018; Reissmann et al. 2017). The patient's self‐perception of oral rehabilitation with fixed dental prostheses should be considered when assessing Oral Health‐related Quality of Life (OHRQoL). OHRQoL can be assessed using the Oral Health Impact Profile‐14 (OHIP‐14), a multidimensional instrument used to assess the subjective perceptions of the esthetics, psychological and functional impacts on oral condition (Hägglin et al. 2007; Locker 2003).

This study assesses and compares the survival of tooth‐ and implant‐supported fixed dental prostheses (including ISCs and ISFPPs) in the posterior jaw region. In addition, this study reports the biological and technical complications and Patient‐Reported Outcomes Measures (PROMs) of two oral prosthetic treatment alternatives. The hypothesis was that survival rates of TSFDPs and implant‐supported fixed dental prostheses (ISCs and ISFPPs) are comparable and that TSFDPs have fewer complications.

## 2 Material and Methods

This study was approved by the Swedish Ethical Review board in Gothenburg (Dnr 2021‐05123).

### 2.1 Design

This retrospective multicenter clinical study was a collaborative effort between two specialist dental clinics in two regions: the Department of Prosthetic Dentistry, Mälar Hospital (Eskilstuna, Sweden) and the Department of Prosthetic Dentistry, Postgraduate Dental Education Center (Örebro, Sweden).

### 2.2 Patient Inclusion

#### 2.2.1 Inclusion Criteria

- Patients referred for prosthetic treatment to the specialist dental clinics and treated between 2009 and 2019 with TSFDP, ISC, or ISFPP
- Patients ≥ 18 years old and with a tooth gap 1–4 posterior to the canine with one or more natural teeth posterior to the tooth gap and treated with either a TSFDPs of 3–5 units, an ISCs, or an ISFPPs with 2–4 units

### 2.3 Data Collection

A retrospective case selection was performed by screening the dental records of all patients treated between 2009 and 2019 with tooth‐ or implant‐supported fixed dental prostheses in two regions at specialist dental clinics. To enable comparison of tooth‐ and implant‐supported FDPs, the tooth‐supported group was matched with approximately an equal number of patients treated with implant‐supported FDPs. All patients treated with TSFDPs in the posterior region and a matching number of patients treated with ISCs or ISFPPs during the same period were invited to participate in clinical and radiological examinations between 2023 and 2025. The aim was to recruit 100 patients in each group (tooth‐ and implant‐supported groups). Patients who fulfilled the inclusion criteria (*N* = 254) received a letter with information about the study and an invitation to participate in the study. Of these, 48 patients with TSFDP and 26 patients with ISC or ISFPP did not respond to the invitations as they either had moved away from the area or were not interested in participating. A total of 180 patients with 238 prostheses agreed to participate and were included. All patients signed an informed consent before examination.

The investigated sample was divided into two groups: tooth‐supported fixed dental prostheses (TSFDPs) group and implant‐supported fixed dental prostheses group (ISCs and ISFPPs). Some patients had both tooth‐supported and implant‐supported prostheses (mixed) (Table 1). Complementary data were retrieved from patient records.

**TABLE 1:** Patients characteristics.

Patients *N* = 180	Only TSFDP group (*n* = 85)	Only ISC and ISFPP group (*n* = 83)	*p* ^a^	Mixed group (*n* = 12)	*p* ^b^
Age, mean (SD)	60.6 (9.2)	49.7 (16.4)	< 0.01	56.3 (17.4)	< 0.01
Women, no. (%)	49 (57.6)	44 (53.0)	0.55	7 (58.3)	0.82
Study center					
Clinic A, no. (%)	43 (50.6)	35 (42.2)	0.27	8 (66.7)	0.22
Clinic B, no. (%)	42 (49.4)	48 (57.8)		4 (33.3)	
Smoker, no. (%)	11 (12.9)	8 (9.6)	0.50	1 (8.3)	0.86
Diabetes, no. (%)	8 (9.4)	9 (10.8)	0.76	1 (8.3)	0.92
Attrition, no. (%)	52 (61.2)	52 (62.6)	0.84	6 (50.0)	0.72
Year of intervention					
2009–13, no. (%)	60 (70.6)	60 (72.3)	0.81	5 (41.7)	0.11
2014–19, no. (%)	25 (29.4)	23 (27.7)		7 (58.3)	

*Note:* Mixed groups include patients with both implant‐ and tooth‐supported prostheses.

Abbreviations: ISC, Implant‐supported‐crown; ISFPP, Implant‐supported fixed partial prosthesis; SD, Standard deviation; TSFDP, Tooth‐supported fixed dental prostheses.

^a^ Calculated with least significant difference (LSD) post hoc test after one‐way ANOVA for age and chi‐squared test or Fisher's exact test when appropriate for the categorical variables comparing only implant vs. only TSFDP groups.

^b^ Calculated with one‐way ANOVA for age and chi‐squared test or Fisher's exact test when appropriate for the categorical variables comparing three groups.

Twelve of the 180 patients, five with TSFDPs and seven with ISFPPs, were not examined clinically due to loss of prosthesis (not in situ). For these patients, only patient records and radiographs were analyzed, and the diagnosis and cause of failure were based on clinical documentation from the treating dentist, patient reports, and examination of the radiographs.

The tooth‐supported fixed dental prostheses (TSFDPs) group included 113 TSFDPs of 3–5 units in the posterior region. The implant‐supported fixed dental prostheses group (ISCs and ISFPPs) included 125 ISCs and ISFPPs in the posterior region.

### 2.4 Survival

Survival was considered when the prosthesis and abutment tooth or implant were in situ. Failure was considered when the prosthesis, abutment tooth, or implant was lost.

### 2.5 Clinical Examination

The clinical examination, performed at follow‐up according to a protocol, included type of fixed prosthesis, position, material of the prosthesis, number and status of supporting teeth or implants, jaw relation, type of antagonist, and number of occluding tooth pairs at delivery of the prosthesis and at follow‐up. In addition, signs of attrition (i.e., visually detectable wearing facets of the incisal edges of the anterior teeth and cusps of the posterior teeth) were recorded. Three prosthodontists performed the clinical and radiographical examinations. A calibration was performed between the examiners. Data were retrieved from patients' records from the date of TSFDPs, implant(s), ISCs, and ISFPPs insertions to the follow‐up appointments. The patients were asked about general medical histories and any complications handled inside or outside the specialist clinic after the insertion of the prostheses.

The examination included a standard periodontal assessment of abutment teeth and implants, which included grading furcation involvement for multi‐rooted teeth, assessing bleeding on probing, and identifying pocket depth > 3 mm (six‐point probing at teeth and four‐point probing at implants) (Rodrigo et al. 2018) using a periodontal probe and a light probing pressure of 20–25 g. When a history of chronic periodontitis was identified, it was recorded for each participant in the tooth‐supported group (Armitage 1999) and according to classification of periodontitis Stage I–II and Stage III (Papapanou et al. 2018). Status of the TSFDPs, ISCs, ISFPPs, implant(s), abutment teeth, prosthesis survival, and biological and technical complications were recorded.

### 2.6 Biological Complications

The recorded biological complications in abutment teeth were caries, marginal or apical periodontitis after TSFDPs insertions, loss of abutment teeth, and gingivitis. Marginal periodontitis was diagnosed and recorded when pocket probing depth was ≥ 6 mm, bleeding on probing was evident and radiographic marginal bone loss was ≥ 3 mm (periodontitis Stage III) (Papapanou et al. 2018). Marginal bone loss (MBL) at abutment teeth was also recorded. The recorded biological complications in implants were mucositis, peri‐implantitis, and marginal bone loss (MBL). Mucositis and peri‐implantitis were diagnosed according to the consensus report of the 2017 World Workshop of the American Academy and European Federation of Periodontology (Berglundh et al. 2018; Heitz‐Mayfield and Salvi 2018; Schwarz et al. 2018).

### 2.7 Technical Complications

The recorded technical complications for TSFDPs were loss of retention, abutment teeth fractures, superficial chip‐off fractures, and extensive ceramic fractures of veneer or crown (i.e., extensive ceramic fracture a fracture requiring repair or change in prosthesis). Technical complications recorded for ISCs and ISFPPs were retention loss (cement or retention screw loosening), superficial chip‐off fractures, extensive ceramic fractures, and loss of screw hole filling.

### 2.8 Radiographical Examination at Follow‐Up

Digital apical and bitewing radiographs were taken at the clinical examination for all abutment teeth and implants in case routine follow‐up radiographs were missing or older than 1 year in the radiograph record programs Dental Eye (version 3.34 Enterprise, Almasoft AB, Stockholm, Sweden) and Life Care Vision (version 2, Tietoevry Corporation Healthcare Welfare Life Care Dental, Finland). This data collection was performed to screen for periapical pathologies and to assess the MBL for abutment teeth in TSFDP group and MBL for the implants in implant‐supported group.

### 2.9 Marginal Bone Level Measurements at Abutment Teeth

All radiographical assessments for marginal bone loss (MBL) at abutment teeth of tooth‐supported group were made using available readable intraoral radiographs taken with bitewing holder at baseline within the first year and at check‐ups at the general dentists between the 5‐year or the 10‐year follow‐up examination included in the study (5 or 10 years depending on the follow‐up time at the examination). The assessments were performed by two calibrated prosthodontists (first author ZA and last author AE) (Hellen‐Halme et al. 2020) using the software programs (the linear measurement) in Dental Eye and IDS7 Version 26.1 (Sectra AB, Linköping, Sweden). As the cement‐enamel junction (CEJ) was not visible in the crowned abutment teeth, the lowest projected edge of the fixed dental prosthesis was used as the reference point. The distance between this reference point and the most apical radiographic marginal bone level on the mesial and distal aspects of the abutment tooth was measured and recorded. The marginal bone level was calculated as the mean of the mesial and distal measurements for each abutment tooth. The marginal bone level was compared between baseline and follow‐up. The change in marginal bone level was calculated and considered a marginal bone loss. The MBL at abutment teeth at the 5‐year and the 10‐year follow‐ups was recorded and reported in categories: 0–1 mm; > 1–2 mm; > 2–3 mm; > 3–4 mm; and > 4 mm.

### 2.10 Marginal Bone Level Measurements at Implants

All patients receiving implant‐supported FDPs at the two specialist dental clinics were offered a routine follow‐up program that included clinical examination and radiographs at 1 and 5 years after treatment. In most cases, radiographs were taken at the department of oral radiology in each clinic. The radiographical assessments were made on intraoral radiographs with periapical projection taken at baseline (loading of the implants) within the first year, at the 5‐year follow‐up, and at the 10‐year follow‐up (5‐ or 10‐year depending on the follow‐up time at the examination). Marginal bone level was measured mesially and distally for each implant as the distance between the implant shoulder/implant‐abutment junction and the first visible bone‐implant contact. The mean marginal bone level was calculated using the mesial and distal measurements for all calculations of bone level changes. Measurements were made using the software Image J version 1.54 g (National Institute of Health, Bethesda, MD, USA). Each radiograph was calibrated for dimensional changes using the inter‐thread distance of the visualized implant in millimeters. All measurements were made by one of the authors (JB), a specialist in oral and maxillofacial radiology, using a dedicated medical workstation for radiologists. Calibration was made between this specialist and two prosthodontists (ZA and AE) before and after the radiographic measurements. The marginal bone level at all implants was measured two times. Whenever it was not possible to make an absolute measurement due to unclear point of reference or inter‐threading, visual assessment was made by comparing the marginal bone level to the same position in other radiographs in the baseline–follow‐up series. MBL at implants was reported using the following categories (Jimbo and Albrektsson 2015): > 0–1 mm; > 1–2 mm; > 2–3 mm; > 3–4 mm; and > 4 mm. In addition, MBL after the first year of implant installation was calculated and documented for each implant according to Albrektsson et al., when MBL was greater than 0.2 mm annually (Albrektsson et al. 1986).

### 2.11 Impact of Treatment on Oral Health‐Related Quality of Life (OHRQoL)

A Swedish version of Oral Health Impact Profile (OHIP‐14) was used to identify treatment alternatives that had the greatest influence on OHRQoL (Hägglin et al. 2007; Locker 2003; Slade 1997). Higher scores indicate worse and lower scores better OHRQoL. The range of scores (0–70) was calculated by summing the result of the answers to the 14 questions in the OHIP‐14. The means of the sums of the OHIP‐14 scores of the two treatment options were compared.

The present article was prepared according to the instructions in Strobe guidelines for observational studies.

### 2.12 Statistical Analysis

Continuous variables were summarized with mean and standard deviation (SD) and compared between groups with one‐way ANOVA and least significance difference (LSD) post hoc test. Categorical variables were summarized with numbers and percentages and compared with Chi‐squared test or Fisher's exact test when appropriate. *p* < 0.05 was regarded as statistically significant and statistical calculations were performed by STATA release 17 (Stata Corp, College Station, TX, USA) and SPSS version 29 (IBM, Chicago, USA).

#### 2.12.1 Survival

Marginal Cox models, adjusting for within patient correlation using a robust variance estimator, were used to evaluate survival for the prosthesis in situ at the follow‐up examination (ISC and ISFPP group vs. TSFDP group). The analysis was performed unadjusted and adjusted for study center (clinic A and clinic B) and patient's age as a linear covariate and smoking (smokers, former, nonsmokers). The proportional hazard assumption was evaluated with the STATA PHTEST command. Due to nonproportional hazard, smoking was included as a stratification variable. Cox regression estimates hazard ratios (HR) with 95% confidence intervals (CI) as association measures. Unadjusted Kaplan–Meier failure plots were used to visualize the survival over time.

#### 2.12.2 Complications

The time‐to‐event analysis described above was not used for complications because the exact time of complication is unknown. Complications were calculated by dividing complications by number of observed events (i.e., complications by the total number of analyzed prostheses). The confidence interval for complications was calculated using the Wilson Score Interval. Complications were analyzed with Poisson regression adjusting for within‐patient correlation using a robust variance adjusting for the same variables as described above. Poisson regression estimates relative risk (RR) as association measures.

#### 2.12.3 Marginal Bone Loss (MBL) at Abutment Teeth and Implants

The radiological measurements of each abutment tooth or implant were analyzed with descriptive statistics at baseline, ≥ 5‐year follow‐up and ≥ 10‐year follow‐up. Descriptive statistics were made by calculating the difference of marginal bone level between baseline and ≥ 5‐year follow‐up, between baseline and ≥ 10‐year follow up and between ≥ 5 year and ≥ 10‐year follow‐ups. The radiographical measurements of MBL at abutment teeth and implants were reported as descriptive categories. For the implants, the unadjusted association between smoking (smokers, former, nonsmokers) and MBL changes from baseline up to ≥ 5‐year follow‐up was analyzed using a random intercept linear mixed model that compensates for the dependency of implants within patients.

#### 2.12.4 Oral Health‐Related Quality of Life

OHIP14 outcome was analyzed with negative binomial regression adjusting for age and study center variables. Negative binomial regression estimates mean ratio as association measures. A mean ratio is a relative mean difference, so a mean ratio of 0.50 is interpreted as the exposed group having half (50%) of the average value of the control group (TSFDP group).

## 3 Results

### 3.1 Patients' Characteristics

The participants included 85 patients treated with only TSFDPs during the inclusion period (2009–2019) and a matched group of 83 patients with only implant‐supported fixed dental prostheses (ISCs and/or ISFPPs). In addition, 12 patients participated who had both types of prostheses (tooth‐supported and implant‐supported) and treated during the same inclusion period. This resulted in the inclusion of 180 patients (Table 1). The TSFDP group included 16 patients who had more than one TSFDP (per patient), and the implant‐supported group included 30 patients with more than one ISC or ISFPP (per patient). Only the patient's age at the time of prosthesis placement differed significantly between the groups (*p* < 0.01) with the TSFDP group having the higher age (11 years higher) (Table 1). The attrition was mild‐to‐moderate in all groups of patients (none had severe attrition).

### 3.2 Characteristics of Prostheses

There was a total of 113 TSFDPs (3–5 units) in 97 (85 + 12) patients supported by 258 abutment teeth (Table 2). Of these abutment teeth, 67 (26%) were non‐vital with root canal filling and 44 of these were provided with post and core. Of the TSFDPs, 30 (27.8%) were cemented with zinc‐phosphate cement (Harvard), 67 (59.2%) with glass ionomer cement (Ketac Cem 3M ESPE), 15 (13.9%) with different brands of resin‐cements: Multilink (Automix Unident), Panavia F, Panavia F2.0 (Kuraray), and one (0.9%) with temporary cement.

**TABLE 2:** Characteristics of the included fixed dental prostheses (FDPs).

FDPs	No. of abutments/implants		No. of units					Total no. of FDPs	Total no. of abutments/implants	Total no. of units
			1	2	3	4	5			
Tooth‐supported		2			65	16	3	84	168	274
		3			0	17	9	26	78	113
		4			0	0	3	3	12	15
	Total				65	33	15	113	258	402
Implant‐supported		1	71					71	71	71
		2		32	21			53	106	127
		3				1		1	3	4
	Total		71	32	21	1		125	181	202

In total, l25 implant‐supported fixed dental prostheses (71 ISCs and 54 ISFPPs) in 95 (83 + 12) patients supported by 181 implants were included (Table 2). Of these, 79 implants were provided with abutments between implants and prosthesis: 70 titanium abutments (37 of them in ISCs), five zirconia abutments, and four titanium bases in ISCs. Table 3 lists the different materials used in TSFDPs, ISCs, and ISFPPs, and Table 4 lists the implant system, diameter of the included implants, and the type of retention used for ISCs and ISFPPs.

**TABLE 3:** Number of included prostheses according to material.

Type of prosthesis	CoCr/p	Ti/p	Ti/a	Gold/p	Zirconia	Lithium disilicate	Aluminum dioxide	Total no. of prostheses
TSFDPs	**91**	**10**	**0**	**1**	**9**	**2**	**0**	113
ISCs	19	15	0	1	31	4	1	71
ISFPPs	34	16	1	0	3	0	0	54
Total ISCs, ISFPPs	**53**	**31**	**1**	**1**	**34**	**4**	**1**	125
TOTAL	144	41	1	2	43	6	1	238

*Note:* The bold values highlight the comparison between the two groups: the tooth‐supported group and the implant‐supported group (ISCs, ISFPPs).

Abbreviations: CoCr/p, Cobalt chromium porcelain; Gold/p, Gold porcelain; ISCs, Implant‐supported crowns; ISFPPs, Implant‐supported fixed partial prostheses; Ti/a, Titanium acryl; Ti/p, Titanium porcelain; TSFDPs, Tooth‐supported fixed dental prostheses.

**TABLE 4:** Implant characteristics of the included ISCs and ISFPPs.

Implant system	No. of ISCs			No. of implants in ISFPPs		
Implant diameter	Narrow	Regular	Wide	Narrow	Regular	Wide
Straumann bone level	1	2	1	0	2	0
Straumann tissue level	2	18	1	7	31	4
Astra Tech TX	6	6	11	7	17	14
Astra Ev Dentsply	0	0	1	1	2	1
Nobel Brånemark (Nobel Biocare)	2	17	2	4	19	0
Nobel CC (Nobel BioCare)	0	1	0	0	0	0
Ankylos (Dentsply Sirona)	0	0	0	0	1	0
Total	11	44	16	19	72	19

Retention type						
Cement‐retained	5	14	7	0	0	0
Screw‐retained	6	30	9	19	72	19

Abbreviations: ISCs, Implant‐supported crowns; ISFPPs, Implant‐supported fixed partial prostheses.

### 3.3 Survival

Figure 1 shows the Kaplan Meier survival analysis at the prosthesis level (unadjusted). The 5‐year cumulative survival rate was 98% (95% CI: 93%–99%) for TSFDPs, ISCs, and ISFPPs. The 10‐year cumulative survival rate was 94% (95% CI: 86%–97%) for the TSFDPs and 93% (95% CI: 86%–96%) for ISCs and ISFPPs (Table 5). There was no statistically significant difference for survival or risk for failure between the tooth‐ and the implant‐supported groups at the prosthesis level, HR 1.02 (95% CI 0.33–3.20) adjusted for study centers, age and smoking (Table 5) after a mean observation time of 120 ± 36 months for TSFDPs and 120 ± 32 months for ISCs and ISFPPs. The minimum and maximum lengths of function for TSFDPs were 24 months and 172 months, respectfully, and the minimum and maximum lengths of function for the implant‐supported fixed dental prostheses were 23 months and 173 months, respectfully (Figure 1). Table 6 shows the risk factors analysis for failure in tooth‐ and implant‐supported groups at the prosthesis level. Table 7 shows analysis of outcome failure of endodontically treated teeth with and without post vs. not endodontically treated teeth and Table 8 shows analysis of caries outcome in endodontically treated teeth with and without post vs. not endodontically treated teeth.

**FIGURE 1:**
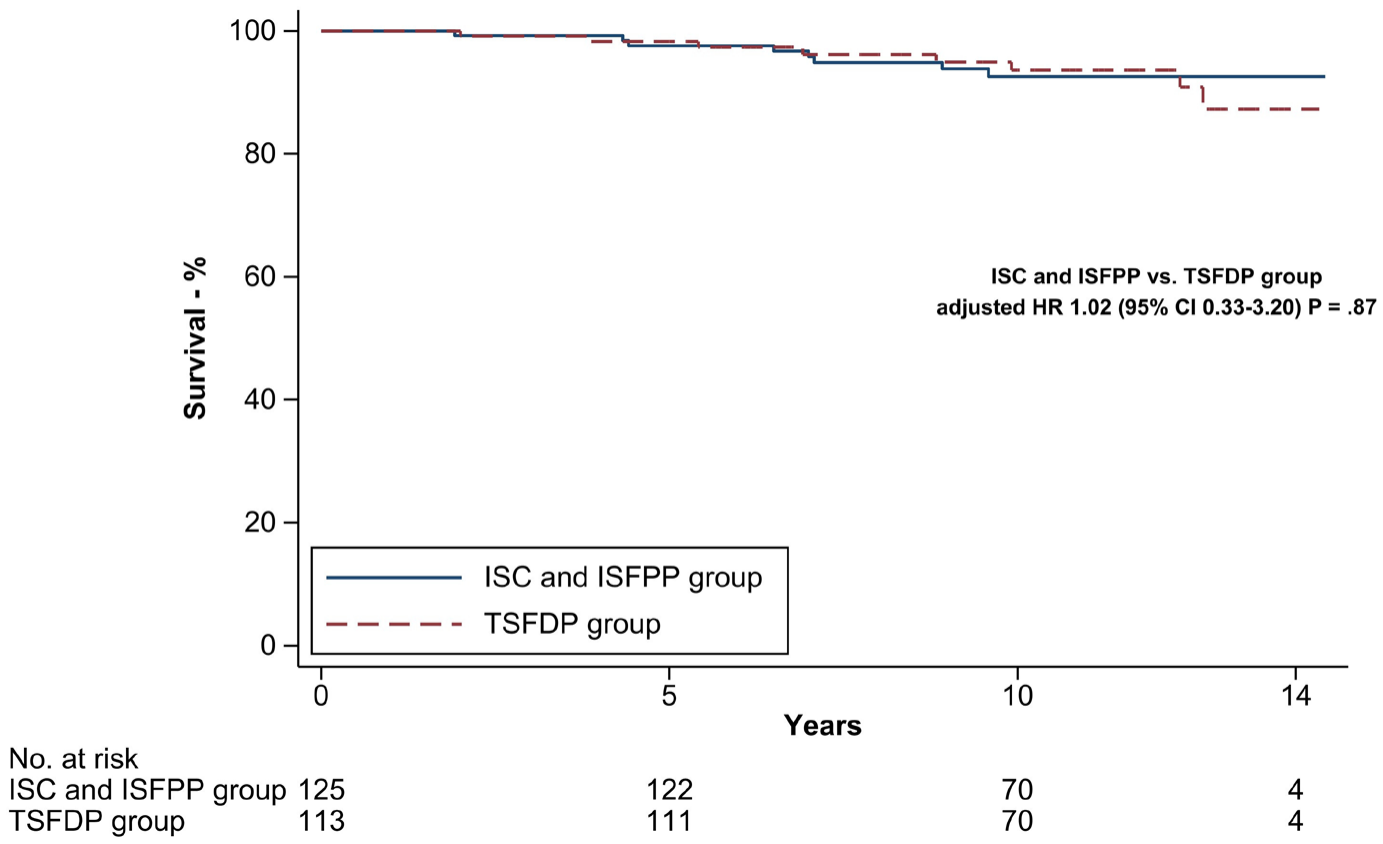
Ilustration of Kaplan Meier survival analysis for tooth‐ and implant‐supported groups.

**TABLE 5:** Survival and failure of tooth‐supported and implant‐supported fixed dental prostheses (FDPs).

238 prostheses (FDPs) 180 patients	FDPs	Failure	Survival		Unadjusted^a^		Adjusted for study centers^a^		Adjusted for study centers, age, and smoking^a^	
	*n*	No. (%)	5 years (95% CI)	10 years (95% CI)	HR (95% CI)	*p*	HR (95% CI)	*p*	HR (95% CI)	*p*
TSFDP group	113	8 (7.1)	98% (93–99)	94% (86–97)	Reference		Reference		Reference	
ISC and ISFPP group	125	8 (6.4)	98% (93–99)	93% (86–96)	0.91 (0.34–2.40)	0.85	0.95 (0.37–2.45)	0.92	1.10 (0.34–3.60)	0.87
Study center										
Clinic A	110	9 (8.2)					Reference		Reference	
Clinic B	128	7 (5.5)					0.46 (0.16–1.30)	0.14	0.51 (0.19–1.36)	0.18
Age, per year									1.01 (0.97–1.04)	0.74
Smoking										
No									Reference	
Former									NA^b^	
Yes									NA^b^	

*Note:* Survival analysed with Cox regression marginal models accounting for repeated prostheses within patients.

Abbreviations: CI, Confidence interval; HR, Hazard ratio; ISC, Implant‐supported‐crown; ISFPP, Implant‐supported fixed partial prosthesis; NA, Not applicable; TSFDP, Tooth‐supported fixed dental prostheses.

^a^ Survival analysis restricted to 5 years follow‐up showed three failures in the ISC and ISFPP group and two failures in the TSFDP group, unadjusted HR 1.35 (95% CI 0.22–8.14) *p* = 0.74, adjusted for study centers HR 1.21 (95% CI 0.22–6.73) *p* = 0.83 and adjusted also for age and smoking HR 1.42 (95% CI 0.23–8.80) *p* = 0.71.

^b^ Due to nonproportional hazards, smoking was adjusted for as a stratification variable in the Cox regression and therefore no hazard ratios could be estimated.

**TABLE 6:** Risk factor analysis for failure at prosthesis level analysed with Cox regression marginal models accounting for repeated prostheses within patients.

All	FDPs	Failure	Unadjusted	
238 prostheses among 180 patients	*n*	No. (%)	HR (95% CI)	*p*
Nonsmokers	208	12 (5.8)	Reference	
Smokers	30	4 (13.3)	2.1 (0.6–7.1)	0.21
Women	127	8 (6.3)	Reference	
Men	111	8 (7.2)	1.3 (0.5–3.5)	0.60
No attrition	90	5 (5.6)	Reference	
Attrition	148	11 (7.4)	1.2 (0.4–3.6)	0.73

TSFDP group	*n*	Failure	Unadjusted	
113 prostheses among 97 patients		No. (%)	HR (95% CI)	*p*
Nonsmokers	98	8 (8.2)	Reference	
Smokers	15	0 (0.0)	NA	NA
Women	65	5 (7.7)	Reference	
Men	48	3 (6.2)	1.0 (0.2–4.2)	0.97
No attrition	40	3 (7.5)	Reference	
Attrition	73	5 (6.8)	0.8 (0.2–3.2)	0.70

ISC and ISFPP group	*n*	Failure	Unadjusted	
125 prostheses among 95 patients		No. (%)	HR (95% CI)	*p*
Nonsmokers	110	4 (3.6)	Reference	
Smokers	15	4 (26.7)	9.8 (2.4–40)	< 0.01
Women	62	3 (4.8)	Reference	
Men	63	5 (7.9)	1.8 (0.4–7.8)	0.46
No attrition	50	2 (4.0)	Reference	
Attrition	75	6 (8.0)	2.0 (0.4–10)	0.40

*Note:* The risk factor analysis was also stratified by TSFDP group and ISC and ISFPP group.

Abbreviations: CI, Confidence interval; HR, Hazard ratio; NA, Not applicable.

**TABLE 7:** Outcome failure of endodontically treated teeth with and without post vs. not endodontically treated teeth.

TSFDP group	*n*	Failure	Unadjusted	
258 abutment teeth among 97 patients		No. (%)	HR (95% CI)	*p*
Not endodontically treated teeth	191	4 (2.1)	Reference	
Endodontically treated teeth	23	1 (4.3)	2.3 (0.4–13.0)	0.35
Endodontically treated teeth with post	44	6 (13.6)	5.3 (1.7–16.8)	< 0.01
Not endodontically treated teeth	191	4 (2.1)	Reference	
Endodontically treated teeth	67	7 (10.4)	4.4 (1.6–12.1)	< 0.01

*Note:* Analysed with Cox regression marginal models accounting for repeated abutment teeth within patients.

Abbreviations: CI, Confidence interval; HR, Hazard ratio.

**TABLE 8:** Caries outcome in endodontically treated teeth with and without post vs. not endodontically treated teeth.

TSFDP group	Teeth	Caries	Unadjusted	*p*
257 abutment teeth among 97 patients	*n*	No. (%)	RR (95% CI)	
Not endodontically treated teeth	191	9 (4.7)	Reference	
Endodontically treated teeth	23	2 (8.7)	1.8 (0.5–6.6)	0.34
Endodontically treated teeth with post	44	5 (11.4)	2.4 (0.9–6.0)	0.058
Not endodontically treated teeth	191	9 (4.7)	Reference	
Endodontically treated teeth	67	7 (10.4)	2.2 (1.0–4.7)	0.038

*Note:* Analysed with Poisson regression marginal models accounting for repeated abutment teeth within patients.

Abbreviations: CI, Confidence interval; RR, Relative risk.

A total of 16 fixed dental prostheses were lost from both tooth‐ and implant‐supported groups. Eight (7.1%) TSFDPs failed (95% CI: 3.6%–13.4%). On the patient level, 8 (8.2%) (95% CI: 4.2%–15.4%) patients in the TSFDP group had a failed prosthesis after a mean follow‐up time of 120 ± 36 months. Table 9 shows the FDPs with failure. Five of the TSFDPs that failed had one or two abutment teeth with post and core (Table 9). For the implant‐supported group, implant loss was 5.5% (95% CI: 3.0%–9.9%). In total, 3 ISCs and 5 ISFPPs (6.4%) (95% CI: 3.3%–12.1%) were recorded as failures which corresponds to (8.4%) (95% CI: 4.3%–15.8%) on the patient level after a mean follow‐up time of 120 ± 32 months.

**TABLE 9:** Failure in tooth‐ and implant‐supported groups.

Tooth‐supported FDPs	Duration in months	No. of RCT	Material	AP or perio‐dontitis	No of teeth with AP or periodontitis	No. of abutment teeth loss	Caries	Post	Cause of failure
34(35)36	25	1	CoCr/p	AP	1	0			FDP fracture
33(34)35	47	1	CoCr/p	AP	1	2	X	1	Caries, AP
34(35)36	66	1	CoCr/p			1	X	1	Fracture
14,15(16)17	84		CoCr/p			1	X		Caries
14(15)16	107	1	Ti/p			2	X		Caries
23(24)25	120	1	CoCr/p			0		1	Removed
43(44)45,46	144	3	CoCr/p	Periodontitis	3	3		3	Periodontitis
13(14,16)17	153	1	CoCr/p			2	X	1	Caries

Implant‐supported FDPs		Implant system		Periodontitis	No. of implants with peri‐implantitis	No. of implant loss	Smoking	Attrition	
34(35)36	24	StraumannTL	CoCr/p	X	1	1	X	X	Periimplantitis
44–45	53	Astra	CoCr/p	X	2	2		X	Periimplantitis
24	54	Astra	Zirconia			0		X	Crown fracture
24–25–26	78	Nobel B	CoCr/p	X	2	1	X		Periimplantitis
16	84	StraumannTL	CoCr/p		1	1	X	X	Periimplantitis
24–25	86	Astra	CoCr/p	X	2	2	X		Periimplantitis
14–15(16)	107	Astra	Ti/p		2	2		X	Periimplantitis
35	115	Nobel B	Zirconia		1	1		X	Periimplantitis

*Note:* Removed = to extend prosthetic treatment due to loss of neighboring tooth.

Abbreviations: AP, apical periodontitis of tooth‐supported FDPs; CoCr/p, cobalt‐chromium porcelain; FDPs, fixed dental prostheses; Nobel B, Nobel Biocare; RCT, root canal therapy; Ti/p, titanium porcelain; TL, tissue level.

### 3.4 Complications

Complications were recorded in 51 (45.1%) TSFDPs (gingivitis not included) and in 42 (33.6%) ISCs or ISFPPs (mucositis not included). There was no statistically significant difference between the tooth‐ and implant‐supported groups for the risk of overall complications, RR 0.75 (95% CI 0.52–1.09) adjusted for center, age, and smoking (Table 10). Table 11 shows the frequencies of all complications, including gingivitis and mucositis, with TSFDPs, ISCs, and ISFPPs.

**TABLE 10:** Complications in the TSFDP group vs. ISC and ISFPP group.

238 prostheses and 180 patients	*n*	Biological complications	Unadjusted		Adjusted 1 (for study centers)		Adjusted 2 (for study centers, age and smoking)	
		No. (%)	RR (95% CI)	*p*	RR (95% CI)	*p*	RR (95% CI)	*p*
TSFDP group	113	28 (24.8)	Reference		Reference		Reference	
ISC and ISFPP group	125	12 (9.6)	0.39 (0.20–0.73)	< 0.01	0.40 (0.21–0.76)	< 0.01	0.42 (0.21–0.83)	0.012

225 prostheses and 168 patients	*n*	Technical complications	Unadjusted		Adjusted 1		Adjusted 2	
		No. (%)	RR (95% CI)	*p*	RR (95% CI)	*p*	RR (95% CI)	*p*
TSFDP group	107	32 (29.9)	Reference		Reference		Reference	
ISC and ISFPP group	118	32 (27.1)	0.91 (0.57–1.44)	0.68	0.90 (0.57–1.43)	0.66	0.88 (0.54–1.44)	0.62

223 prostheses and 166 patients	*n*	Chip‐off fracture	Unadjusted		Adjusted 1		Adjusted 2	
		No. (%)	RR (95% CI)	*p*	RR (95% CI)	*p*	RR (95% CI)	*p*
TSFDP group	106	22 (20.7)	Reference		Reference		Reference	
ISC and ISFPP group	117	18 (15.4)	0.74 (0.40–1.36)	0.33	0.71 (0.39–1.31)	0.28	0.77 (0.41–1.45)	0.42

224 prostheses and 167 patients	*n*	Extensive ceramic fractures	Unadjusted		Adjusted 1		Adjusted 2	
		No. (%)	RR (95% CI)	*p*	RR (95% CI)	*p*	RR (95% CI)	*p*
TSFDP group	106	12 (11.3)	Reference		Reference		Reference	
ISC and ISFPP group	118	2 (1.7)	0.15 (0.03–0.68)	0.014	0.15 (0.03–0.71)	0.016	0.15 (0.04–0.58)	< 0.01

238 prostheses and 180 patients	*n*	Overall complication	Unadjusted		Adjusted 1		Adjusted 2	
		No. (%)	RR (95% CI)	*p*	RR (95% CI)	*p*	RR (95% CI)	*p*
TSFDP group	113	51 (45.1)	Reference		Reference		Reference	
ISC and ISFPP group	125	42 (33.6)	0.74 (0.53–1.05)	0.093	0.74 (0.53–1.05)	0.097	0.75 (0.52–1.09)	0.13

*Note:* Analysed with Poisson regression marginal models accounting for repeated prostheses within patients.

Abbreviations: CI, Confidence interval; ISC, Implant‐supported crown; ISFPP, Implant‐supported fixed partial prosthesis; RR, Relative risk; TSFDP, Tooth‐supported fixed dental prostheses.

**TABLE 11:** Complications of tooth‐ and implant‐supported FDPs up to 14 years.

Complications tooth‐supported group	Unit level^a^	95% CI boundaries%^b^	Prosthesis level^a^	95% CI boundaries%^b^	Patient level^a^	95% CI boundaries%^b^
Chip‐off fracture	22 (5.8)	3.9–8.7	22 (20.7)	14.1–29	20 (22.4)	15–32
Extensive ceramic fracture	14 (3.7)	2.2–6.1	12 (11.3)	6.6–18.8	10 (11)	6–12
History of loss of retention			6 (5.3)	1.5–9.3		
Fracture without repair			21 (18.6)	12.5–26.8		
Fracture with repair			0			
Caries			14 (12.4)	7.5–19.7	13 (13.4)	8.0–21.6
Gingivitis			47 (43.5)	34.6–52.9	40 (43.4)	33.8–53

	Abutment level^a^	95% CI boundaries%^b^	Prosthesis level^a^	95% CI boundaries%^b^	Patient level^a^	95% CI boundaries%^b^
Apical periodontitis	15 (5.8)	3.6–9.4				
Marginal periodontitis	12 (4.6)	2.6–7.9	5 (4.4)	1.9–9	4 (4.1)	1.6–10
Abutment tooth loss	11 (4.3)	2.4–7.5				
RCT through TSFDP	7 (2.7)	0.7–4.7				
No. of teeth with caries	16 (6.2)	3.9–9.8				
Failure (not in situ)			8 (7.1)	3.6–13.4	8 (8.2)	4.2–15.4

Complications implant‐supported group	Unit level^a^	95% CI boundaries%^b^	Prosthesis level^a^	95% CI boundaries%^b^	Patient level^a^	95% CI boundaries%^b^
Chip‐off fracture	23 (12.3)	8.3–17.8	18 (15.4)	10.0–23.0	17 (19.3)	12.4–28.8
Extensive ceramic fracture	2 (1)	0.3–3.8	2 (1.7)	0.5–6.0	2 (2.2)	0.6–7.8
History of screw loosening			11 (8.8)	5.0–15.1	11 (11.6)	6.6–19.6
Fracture without repair			11 (8.8)	5.0–15.1		
Fracture with repair			1 (0.8)	0.1–4.4		
Loss of screw hole filling			4 (3.2)	1.3–7.9		
Mucositis			33 (28.0)	20.7–36.7	26 (29.5)	21.0–39.8

	Implant level^a^	95% CI boundaries%^b^	Prosthesis level^a^	95% CI boundaries%^b^	Patient level^a^	95% CI boundaries%^b^
Peri‐implantitis	16 (8.8)	4.7–12.6	12 (8.0)	4.4–14.1	12 (12.6)	5.8–18.3
MBL > 0.2 mm annually	20 (11)	7.3–17			13 (13.6)	8–22
Implant loss	10 (5.5)	3.0–9.9	7 (5.6)	2.7–11	7 (7.4)	3.6–14.4
Failure (not in situ)			8 (6.4)	3.3–12.1	8 (8.4)	4.3–15.8

*Note:* History of screw loosening was recorded in 5 ISCs and 6 ISFPPs.

Abbreviations: CI, Confidence interval; MBL, Marginal bone loss; RCT, Root canal therapy.

^a^ Frequency and percent: *n* (%).

^b^ Wilson score interval.

### 3.5 Biological Complications

Twenty‐eight (24.8%) TSFDPs had biological complications (gingivitis not included). Five initial vital abutment teeth had apical periodontitis at the follow‐up examination and 10 abutment teeth with root canal filling had apical periodontitis (a total of 15 abutment teeth with apical periodontitis). A probing pocket depth > 3 mm at prosthesis level was recorded in 42 (39.6%) TSFDPs (95% CI: 30.8%–49.1%), and furcation involvement greater than Grade I was present in abutment teeth in 4 (3.8%) TSFDPs (95% CI: 1.5%–9.3%). A history of chronic periodontitis (Stage I–II) was recorded in 37 (38%) patients in the TSFDP group before placement of the FDP. Three patients were diagnosed with marginal periodontitis Stage III at the follow‐up examination and one patient at the time of prosthesis failure (tooth loss) (Table 11).

In the implant group, 12 (9.6%) ISCs and ISFPPs had biological complications (mucositis not included). There were statistically significantly fewer biological complications in the implant‐supported prostheses group, adjusted RR 0.42 (95% CI: 0.21–0.83) (Table 10).

#### 3.5.1 Marginal Bone Loss (MBL) at Abutment Teeth

Digital radiographs of 258 abutments were examined and assessed. Radiographs for 256 abutment teeth were considered as readable for bone level assessment. MBL was categorized for all teeth (Table 12). Most of the abutment teeth (*n* = 234) had an MBL between 0 and 1 mm.

**TABLE 12:** Marginal bone loss (MBL) at abutment teeth and implants, number and (%).

MBL	Abutment teeth 5‐year follow‐up	Abutment teeth 10‐year follow‐up	MBL	Implants 5‐year follow‐up	Implants 10‐year follow‐up
0–1 mm	76 (95)	158 (89)	0 mm	84 (48.6)	57 (45.3)
			> 0–1 mm	67 (38)	44 (34.9)
> 1–2 mm	3 (3.7)	14 (7.9)	> 1–2 mm	8 (4.6)	15 (11.9)
> 2–3 mm	1 (1.3)	2 (1)	> 2–3 mm	4 (2.3)	2 (1.6)
> 3–4 mm	0	0	> 3–4 mm	6 (3.5)	4 (3.2)
> 4 mm	0	2 (1)	> 4 mm	4 (2.3)	4 (3.2)
Total no. abutment teeth	80 (100)	176 (100)	Total no. implants with MBL	89 (51.4)	69 (54.7)

#### 3.5.2 Marginal Bone Loss (MBL) at Implants

At baseline, the marginal bone level of the 181 included implants in the study was well documented from digital radiographs except for two implants. Radiographs were available for 173 implants at the 5‐year follow‐up (i.e., between 5 and < 9 years) and for 126 implants at the 10‐year follow‐up (i.e., between 9 and 14 years). MBL for the implants lost due to peri‐implantitis were recorded as ≥ 4 mm.

At the 5‐year follow‐up (i.e., between 5 and 9 years), 84 (48.6%) implants had no MBL. At the 10‐year follow‐up (i.e., between 9 and 14 years), 57 (45.3%) implants had no MBL. At both follow‐ups, MBL was recorded as one of five categories (Table 12). Table 9 shows the time in function of lost implants. All implant losses occurred before the 10‐year follow‐up. Smokers had statistically significantly higher MBL than former smokers and nonsmokers (*p* < 0.01) at the 5‐year follow‐up. Thirteen patients with 20 implants (11%) had MBL > 0.2 mm annually after the first year (Table 11). Of these patients, 8 (with 14 implants) were diagnosed with peri‐implantitis. Thus, the success of implants in this study was 89% after 5–14 years on implant level according to the suggested success criteria (Albrektsson et al. 1986).

### 3.6 Technical Complications

Thirty‐two (29.9%) TSFDPs had complications. In the implant group, technical complications were recorded in 32 (27.1%) ISC and ISFPPs. There was no statistically significant difference between the tooth‐ and implant‐supported prostheses groups for the risk of technical complications, adjusted RR 0.88 (95% CI: 0.54–1.44) (Table 10).

#### 3.6.1 Ceramic Fractures

Chip‐off fractures were recorded in 22 (20.7%) TSFDPs. Of these, 17 TSFDPs were made of cobalt‐chromium/porcelain (CoCr/p) and 5 of zirconia (Table 10). Extensive ceramic fractures were recorded in 12 (11.3%) TSFDPs. Of these, 10 were made of CoCr/p, and 2 were of titanium/porcelain (Ti/p). In the implant‐supported group, 18 (15.4%) of the prostheses had chip‐off fractures: 10 ISCs (five zirconia, two Ti/p, one CoCr/p, one gold/p, and one aluminum oxide), and 8 ISFPPs (five Ti/p and three CoCr/p). Extensive ceramic fractures were recorded in 2 prostheses (1.7%)–one ISC made of zirconia and one ISFPPs made of Ti/p.

There was no statistically significant difference in chip‐off fractures at the prosthesis level between the implant‐ and the tooth‐supported groups RR 0.77 (95% CI: 0.41–1.45), but there were statistically significantly fewer extensive ceramic fractures in the implant‐supported group RR 0.15 (95% CI: 0.04–0.58) (Table 10).

### 3.7 Oral Health‐Related Quality of Life

Table 13 shows the mean scores of patients with TSFDPs, ISCs, and ISFPPs and the mixed group of patients that have both TSFDP and ISC or ISFPP. There was a statistically significant difference between the two main groups, tooth‐supported and implant‐supported, but this was not of clinical relevance as both TSFDP and ISC/ISFPP groups had low OHIP‐14 mean scores (i.e., < 5) and even the mixed group mean score was < 5. Table 13 shows the results with and without adjusting for study center (clinic A and clinic B) in the two regions and by age. Both treatments showed low mean OHIP‐14 scores, indicating a good OHRQoL.

**TABLE 13:** OHIP‐14 in patients with tooth‐ and implant‐supported fixed dental prostheses analysed with negative binomial regression.

168 patients	Patients	OHIP‐14	Unadjusted		Adjusted for study centers		Adjusted for study centers and age	
	n	Mean (SD)	Mean ratio (95% CI)	p	Mean ratio (95% CI)	p	Mean ratio (95% CI)	p
Tooth‐supported group	80	4.8 (6.7)	Reference		Reference		Reference	
Implant‐supported group	76	3.0 (4.3)	0.62 (0.36–1.05)	0.078	0.62 (0.36–1.06)	0.079	0.57 (0.33–0.99)	0.046
Mixed group	12	3.5 (3.6)	0.72 (0.26–2.03)	0.54	0.72 (0.26–2.02)	0.53	0.74 (0.26–2.07)	0.56

*Note:* Mixed group: Patients with both tooth‐ and implant‐supported fixed prostheses. A mean ratio is a relative mean difference and a mean ratio of 0.50 interprets as the implantat group has half (50%) of the average value in the tooth‐supported group.

Abbreviations: CI, Confidence interval; SD, Standard deviation.

## 4 Discussion

### 4.1 Survival

The cumulative 5‐year survival rate for both tooth‐ and implant‐supported fixed dental prostheses was 98% and the 10‐year survival rates were 94% and 93%, respectively. The survival rates decreased after 10 years. These results are comparable to Walton's results for fixed dental prostheses in the posterior region and the results for TSFDPs from earlier long‐term studies after 5 years (93.3%–100%) and after 10 years (84.4%–89.2%) (Bischof et al. 2024; De Backer et al. 2008; Pjetursson et al. 2007, 2015; Walton 2015; Wolleb et al. 2012). The results in the present study resemble the results in systematic reviews, where ISCs and ISFPPs have an estimated survival rate of 96.6%–99% after 5 years and 89.4%–100% after 10 years (Bischof et al. 2024; Hjalmarsson et al. 2016; Jung et al. 2012; Pjetursson et al. 2007, 2012; Wolleb et al. 2012). Two recent studies have reported cumulative survival rate of long and medium span TSFDPs: 90.1% after 5 years; 74.4%–77.6% after 10 years; and 52% after 20 years (Alsterstal‐Englund et al. 2021; Hawthan et al. 2023). These results were somewhat lower than the results of the present study, a difference that might be attributed to different inclusion criteria and the number of units and position of TSFDPs (Alsterstal‐Englund et al. 2021; Hawthan et al. 2023).

### 4.2 Biological Complications TSFDPs

In the present study, the biological complications were statistically higher in the TSFDPs group than in the ISCs and ISFPPs group (*p* = 0.012); this may be due to the higher number of abutment teeth (*n* = 258) examined and included in comparison to the implants (*n* = 181). Eight (7.1%) TSFDPs failed: 4 of 8 TSFDPs due to abutment tooth loss (4.3%), because of secondary caries. Caries was recorded in 16 (6.2%) of the 258 abutment teeth; this finding is higher than the reported 5‐year results in a follow‐up study and a systematic review, which reported a caries rate in the abutment teeth of 0.4% and 4.8%, respectively (Pjetursson et al. 2007; Wolleb et al. 2012). However, the prevalence of caries in the present study was slightly lower than that reported in long‐term studies, where caries rate was reported to be 8%–10.6% (Bischof et al. 2024; Karlsson 1986). Endodontically treated abutment teeth (pretreated) with or without post and core showed higher risk of caries complication and failure compared to vital abutment teeth in the present study. This finding agrees with the results of Bischof et al. that reported a higher probability of complication in endodontically treated abutment teeth in tooth‐supported FDPs (Bischof et al. 2024).

The present study reports apical periodontitis to be 5.8% and marginal periodontitis to be 4.6% after a mean observation period of 10 years, a finding similar to other studies and a systematic review which reported a 2.9%–6.3% loss of vitality or pulp necrosis and apical periodontitis (Al‐Manei et al. 2023; Bischof et al. 2024; Karlsson 1986; Randow et al. 1986; Wolleb et al. 2012). Marginal periodontitis in the present study was lower than in a recent study (Bischof et al. 2024).

### 4.3 Biological Complication of ISCs and ISFPPs

Seven of eight of the ISCs and ISFPPs failures were because of implant losses due to peri‐implantitis (2 ISCs and 5 ISFPPs). Implant loss was 5.5% at the implant level and 7.4% at the patient level. These results compare favorably with the results of a systematic review and a long‐term follow‐up study (Salvi and Zitzmann 2014; Schmidlin et al. 2010). In the present study peri‐implantitis was 8.8% at the implant level and 12.6% at the patient level. The prevalence of peri‐implantitis in the present study was slightly higher compared to a recent long‐term study, which included a cohort of patients who had been successfully treated for periodontal disease and were enrolled in a supportive periodontal care program (Bischof et al. 2024). The patients in the present study were controlled with a clinical examination 1 year and 5 years after insertion of the ISC or ISFPP as routine follow‐up examination and thereafter they were put on a recall program every 5 years. However, not all patients participated in the program. Only patients treated for periodontitis had supportive periodontal care. According to a review, the biological complications in the implant‐supported group in the present study compared favorably to studies published after 2000 (Pjetursson and Heimisdottir 2018). Derks and Tomasi reported a 19%–65% prevalence of mucositis (Derks and Tomasi 2015), which agrees with the results of the present study, where 29.5% of patients were diagnosed with mucositis. In the present study, peri‐implantitis was recorded in 8.8% of implants as compared to the 14.5%–45% of peri‐implantitis reported in the study by Derks et al. (Derks et al. 2016). In the study by Derks et al., the prosthetic treatment was performed by general practitioners whereas the prosthetic treatment in the present study was performed by prosthodontists at specialist dental clinics in two regions. However, both studies included patients with and without a history of periodontitis (Derks et al. 2016). In a recent systematic review, the prevalence of peri‐implantitis was 19.5% at the implant level and 12.5% at the patient level (Diaz et al. 2022). In the present study, prostheses in both the tooth‐ and implant‐supported groups mainly failed because of biological complications.

### 4.4 Marginal Bone Loss (MBL) at Abutment Teeth

In the present study, the MBL at the abutment teeth for the TSFDP group was small for most abutment teeth (*n* = 234/256) 0–1 mm. This finding confirms the results from a recently published study (Pol et al. 2022).

### 4.5 Marginal Bone Loss (MBL) at Implants

The MBL was small for most implants, and the results are favorably comparable to the results of two previous studies (Derks et al. 2016; Eliasson et al. 2006). The MBL at the implants at ≥ 5‐year was higher in smokers than in nonsmokers and former smokers. This finding agrees with the results of a systematic review (Veitz‐Keenan 2016). These results should be interpreted with caution because in the present study only 9.6% of the patients with implants were smokers. In this study implant success was 89% up to 14 years posttreatment (on implant level) according to the suggested success criteria (Albrektsson et al. 1986).

### 4.6 Technical Complications

The prevalences of technical complications for the TSFDP group and of the implant group (ISCs and ISFPPs) were approximately at the same level (29.9% and 27.1%, respectively) despite the heterogenicity of the material and different number of units. The chip‐off fractures at prosthesis level were in 20.7% of TSFDPs and in 15.4% of ISCs and ISFPPs. Extensive ceramic fracture was found in 11.3% of TSFDPs and in 1.7% of ISCs and ISFPPs. This difference in extensive ceramic fracture may be because the tooth‐supported group had TSFDPs with longer span (3–5 units) than the prostheses in implant‐supported group (1–4 units). In the present study, chip‐off fractures were the most common technical complication for metal‐ceramic prostheses. However, the results are comparable to the results of a recent study reporting chip‐off fracture in 20.2% of TSFDPs and 13.9%–15.8% of ISCs and ISFPPs (Bischof et al. 2024). A 5‐year follow‐up study reported a very low loss of retention for TSFDPs, 0.8% (Wolleb et al. 2012). The loss of retention for TSFDPs in the present study was 5.3%, slightly lower than the 7.5% reported in another study but higher than the 0.8% as previously reported (Walton 2015; Wolleb et al. 2012). The prevalence of screw loosening for ISCs and ISFPPs was 8.8%, which is higher than the 3.6%–5.2% in ISCs in another study (Rabel et al. 2018) and lower than the 33% of screw loosening in ISCs reported by Walton (Walton 2015). The prevalence of technical complications of TSFDPs was slightly higher in comparison to the study by Alenezi et al., who reported technical complications in 24.6% of TSFDPs in the posterior region after a mean observation period of 92.8 ± 82 months (Alenezi and Aloqayli 2023).

There was no statistically significant difference for the overall complications rate in the present study for TSFDPs (45.1%) and the implant‐supported prostheses group (33.6%) at the prosthesis level. This finding differs from the reported complications in a systematic review, where 15.7% of patients with TSFDPs and 38% of patients with ISFPPs had complications (Pjetursson et al. 2007). In the present study, the prevalence of TSFDPs without complications was 54.9% and the corresponding value for ISCs and ISFPPs was 66.4%. The results in the present study were lower than the data from Walton, 76.4% for TSFDPs and 83.2% for ISCs without complications (i.e., complications that did not require any retreatment) (Walton 2015). The comparison of outcomes between studies may be difficult and problematic due to variations in patient inclusion, materials and production techniques of the prostheses, and the methodology for reporting complications (Pjetursson et al. 2007; Walton 2015). The complications reported in the present study included chip‐off fractures that did not require retreatment.

### 4.7 OHRQoL

OHIP‐14 revealed low scores for patients with both tooth‐ and implant‐supported fixed dental prostheses. This finding was in accordance with studies reporting no difference in the impact of single tooth implant and three units TSFDP on improvement of OHRQoL (Ozhayat and Gotfredsen 2020; Park et al. 2016). Most studies reporting on OHRQoL using OHIP‐14 or ‐49 evaluate prosthetic treatment of fully edentulous patients (Reissmann et al. 2017). In the present study, partially edentulous patients were evaluated with OHIP‐14; however, the sensitivity of the instrument to capture the impairment of partially edentulous patients treated with tooth‐ or implant‐supported fixed prostheses is questioned (Ozhayat and Gotfredsen 2020).

### 4.8 Limitations

- Retrospective studies have several limitations such as lack of randomness in treatment and material selection of fixed dental prostheses and possible lack of data in patients' records
- The number of all‐ceramic fixed dental prostheses in this study was smaller compared with metal ceramic (11 vs. 102 for TSFDPs and 39 vs. 84 for ISCs and ISFPPs group). Metal‐ceramic material (earlier mostly high precious alloy) used to be the “gold standard” for TSFDPs and ISFPPs. Bischof et al. reported fewer all‐ceramic fixed dental prostheses with long follow‐up time (Bischof et al. 2024). Therefore, we did not compare the different materials of prostheses in this study as the number of all‐ceramic fixed dental prostheses in both groups was limited. The heterogeneity of materials and the variation in the number of units can affect the clinical outcomes and may limit the generalizability of the findings
- Another possible limitation is the lack of regular registrations of plaque index
- MBL at the implants was assessed radiographically without the use of fully standardized radiographic holders for all patients, a methodological limitation that may affect the precision of marginal bone level measurements between examinations; however, all radiographs were taken using an Eggen radiographic holder or a similar device for bitewing radiographs. Peri‐implant bone loss is a commonly reported variable in the implant literature (Pol et al. 2018), but bone loss around teeth is rarely reported (Pol et al. 2018). A limitation in assessing the MBL around abutment teeth in the tooth‐supported group was that the normal reference point (CEJ) was hidden by the abutment crown. Furthermore, calibration of radiographs for the abutment teeth was not possible as for the implants
- Finally, this study may be affected by the absence of values for pretreatment OHIP scores and missing (OHIP‐14) values for patients who lost their FDPs and were not examined clinically

The hypothesis that the survival of TSFDPs and ISCs or ISFPPs is comparable was accepted, but the hypothesis that TSFDPs have fewer complications was rejected.

## 5 Conclusions

Treatment performed in specialist dental clinics by dental specialists showed the following:

- Tooth‐ and implant‐supported fixed dental prostheses had high survival rates after 5 and 10 years
- Tooth‐supported fixed dental prostheses had more biological complications than implant‐supported fixed dental prostheses
- Chip‐off fracture was the most frequent technical complication in both groups
- Treatment of small tooth gaps in the posterior region with tooth‐ or implant‐supported fixed dental prostheses showed a good patient‐reported effect on OHRQoL

## Author Contributions

**Zahra Athab Abduljabbar:** conceptualization, methodology, software, formal analysis, project administration, writing – review and editing, writing – original draft, data curation, investigation, visualization, validation, funding acquisition, resources. **Anders Magnuson:** formal analysis, methodology, writing – review and editing, data curation. **Lars Hjalmarsson:** conceptualization, supervision, resources, writing – review and editing, methodology, investigation. **Victoria Franke Stenport:** conceptualization, writing – review and editing, methodology, supervision, investigation, visualization. **Alf Eliasson:** conceptualization, methodology, software, data curation, supervision, investigation, writing – review and editing, visualization, validation, formal analysis, project administration, resources. **Jir Barzangi:** writing – review and editing, methodology, visualization, software, data curation, conceptualization, formal analysis.

## Funding

This work was supported by Center for Clinical Research Sörmland Uppsala University.

## Conflicts of Interest

The authors declare no conflicts of interest.

## Supporting information


**Appendix S1:** STROBE Statement—Checklist of items that should be included in reports of *cohort studies*.

## Data Availability

The data that support the findings of this study are available on request from the corresponding author. The data are not publicly available due to privacy or ethical restrictions.
